# International Child Abduction: The Complexities of Forensic Psychiatric Assessments Before the Hague Convention

**DOI:** 10.3389/fpsyt.2021.654634

**Published:** 2021-07-09

**Authors:** Mitesh Patel, Shawn Baldeo, Pip Swartz, Graham Glancy

**Affiliations:** ^1^Division of Forensic Psychiatry, Department of Psychiatry, University of Toronto, Toronto, ON, Canada; ^2^Psychiatry Resident, PGY-3, University of Toronto, Toronto, ON, Canada; ^3^Faculty of Law, University of Toronto, Toronto, ON, Canada

**Keywords:** Hague convention, international child abduction, article 13, child custody rights, parental alienation syndrome, forensic psychiatry

## Abstract

The Hague Convention is an international intergovernmental agreement that facilitates the return of abducted children to lawful parents across international borders. Children may not be returned if it can be established that the return would result in harm to the child. Forensic psychiatrists may be called upon to provide an expert opinion regarding the potential harm to come to a child, as well as various other psycholegal issues. We discuss interpretations and precedents regarding this law and the possible contributions of forensic psychiatrists. We also discuss two hybridized case examples involving international child abduction and proceedings before the Hague Convention. We will discuss issues that arose after psychiatric evaluations in each case.

## Introduction

The Hague Convention assists member nations in addressing international child abduction (ICA). Forensic psychiatrists may be requested to provide expert witness testimony in such cases. This presents various ethical and medico-legal challenges. Two case examples are provided to highlight the complexities inherent to applications before the Hague Convention and psycholegal assessments.

### International Child Abduction

ICA occurs when a parent, guardian or other person with lawful care of a child removes that child from a country without the permission or legal authority of a parent or guardian who has full or joint custody rights (1).

In February 2019, there were 187 cases of ICA managed by Global Affairs Canada. In 2015, there were 2,730 applications before the Hague Convention, of which 102 included Canada. The total number of cases involving ICA in Canada are estimated to at least double if these were to include non-convention cases, which are not captured in statistics involving the Hague Convention (2).

ICA has a profound impact on the child. Freeman (3) postulates that the immediate sequelae may include shock from the abduction, loss of a parent and the sudden need to adapt to a new environment. Aside from the immediate shock, abducted children may suffer significant psychological sequelae, including social disorders, adjustment disorders and severe psychological trauma (3).

### The Hague Convention

**Figure 1:**
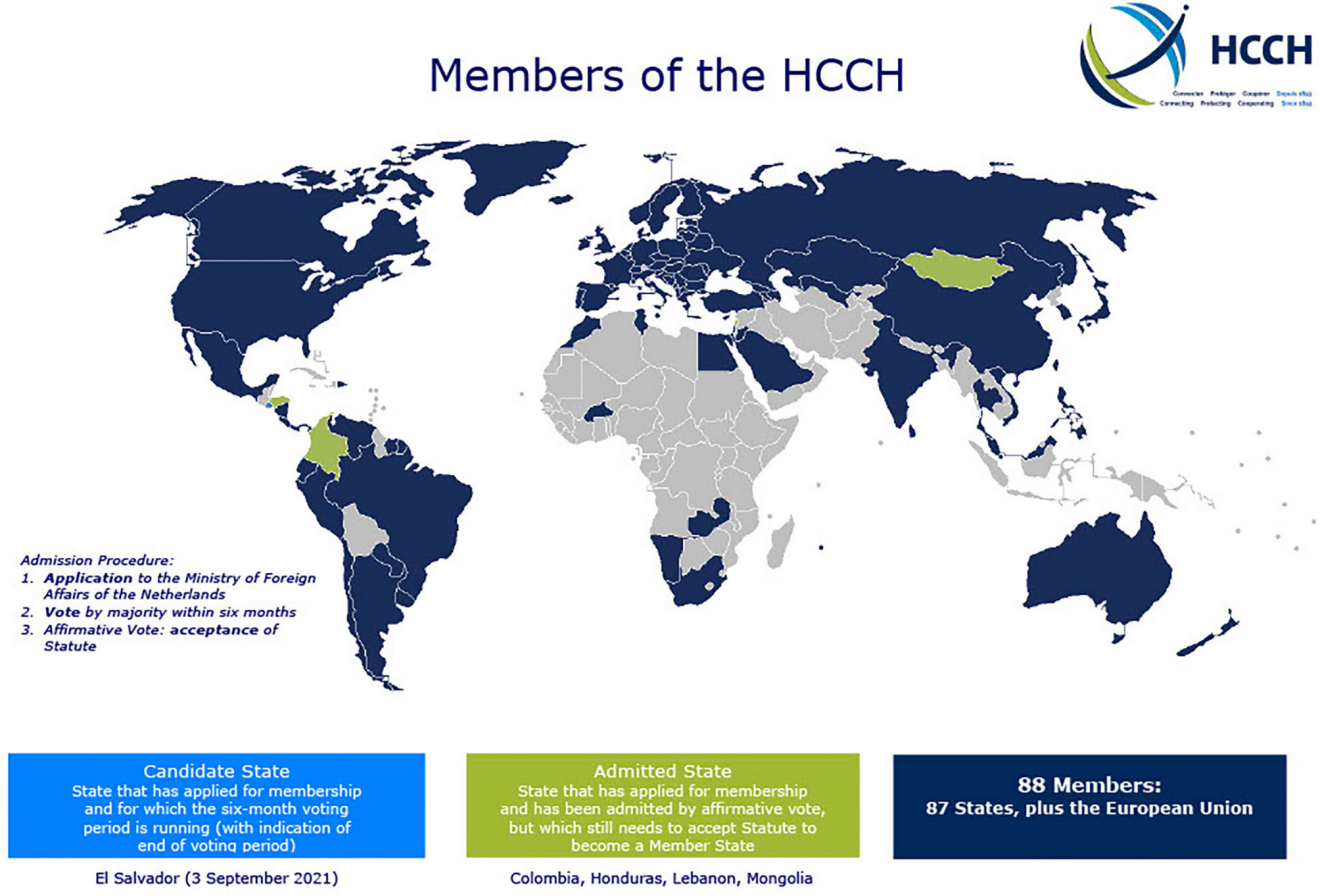
Signatories of the International Hague Convention. Retrieved from http://www.hcch.net on May 15, 2020.

ICA cases can be addressed through the Hague Convention. The Hague Convention is an intergovernmental agreement designed to ensure the timely resolution of abduction matters. As of 2019, there were over 100 signatories to the Convention (see Figure 1).

The objective of the Hague Convention is to secure the prompt return of a child wrongfully removed or retained in any contracting state or country. A court decision under the Convention may order the return of a child to the country of habitual residence (2). The Convention is not designed to manage issues of custody or access, but rather to return the child to the country of origin. Any subsequent custody issues are managed by local law and related procedures in the home jurisdiction (4).

### Exceptions to the Hague Convention

While the purpose of the Convention is to return children to their habitual residence, there are circumstances in which exceptions may be made. Among these is what is known as the “grave risk exception” (5). Article 13(1)(b)10 of the Hague Convention provides:

Notwithstanding the provisions of the preceding Article (12), the judicial or administrative authority of the requested State is not bound to order the return of the child if the person, institution or other body which opposes its return establishes that… there is a grave risk that his or her return would expose the child to physical or psychological harm or otherwise place the child in an intolerable situation (6).

Article 13 notes the judicial or administrative authority may also refuse to order the return of the child if the child objects to being returned and has attained an age and degree of maturity for which it is appropriate to take account of their views (7). The governing body may take into account information relating to the social background of the child, as well as information gleaned from the child's habitual residence. These exceptions often lead to adversarial challenges between parents and between the parents and the child (8). To determine whether the child meets criteria for these exceptions, a forensic mental health assessment may be sought.

## Case Vignettes

We present two hybridized case vignettes based upon clinical case examples of children who were assessed by a dually qualified Canadian Child and Adolescent Forensic Psychiatrist in relation to potential repatriation to a home country under the Hague Convention. Consent was obtained from each child to present aspects of their cases with changes made so as to not identify the children or families involved.

### Case 1: Antoine

Antoine is a 13-year-old male who was born in the United States (US) and brought to Ontario, Canada by his mother, who was a joint Canadian-American citizen, for a 2-week holiday period. His mother and father had previously divorced in the US where Antoine had been raised under the legal custody of both of his parents. Upon the completion of the holiday, the father was informed by the mother that the child would remain with her in Canada. An application was commenced under the Hague Convention for the return of the child to the US.

Antoine was appointed legal counsel in Ontario. He made various allegations of childhood physical and emotional abuse from his father. Antoine's legal counsel retained a Forensic Psychiatrist to provide an expert opinion as to whether Antoine was capable of making decisions regarding his country of residence, and to detail the potential harm that may come to him should he be returned to the US.

As per his psychiatric assessment, Antoine was determined to be a “mature minor,” given that he was able to understand and appreciate the nature and consequences of his decisions, particularly those related to determining his country of residence. Furthermore, Antoine's allegations regarding childhood abuse and severe physical discipline were explored in detail and substantiated by evidence provided through Emergency Department records and child welfare investigation reports.

Antoine also informed the evaluator that he had “come out” and identified as gay over the course of his stay in Canada. He stated that he feared a return to his father's home, as his father was a member of a conservative religious group that would not permit him to freely express his sexual identity.

Antoine was noted to suffer from significant depressive symptoms. He met criteria for Major Depressive Disorder as per the Diagnostic and Statistical Manual version 5 (DSM-5). The psychiatric assessment was provided to the courts by Antoine's legal counsel and this in turn was also provided to Antoine's father's counsel.

Antoine's father's counsel enlisted a registered social worker (RSW) to provide an opinion regarding the forensic psychiatric report and the opinions expressed therein. This RSW was identified as an expert in “Parental Alienation Syndrome” (PAS) by the father's legal counsel. PAS was defined as a constellation of symptoms resulting from “brainwashing” the child by one parent resulting in the child's subsequent vilification of the target parent (9).

The RSW provided an opinion that Antoine's requests were not independent and should not be considered by the courts due to alleged “brainwashing” by his mother. The RSW did not directly interview Antoine but did review available collateral information and the psychiatric assessment that was completed. The RSW also concluded that the impressions and diagnoses provided by the forensic evaluation were severely limited, given that the child was “brainwashed.” The RSW offered a diagnosis of PAS in their assessment.

In the court's ruling, it was determined that the child could pursue protections through local jurisdictional entities in the US. Ultimately, Antoine was returned to the custody of his father in the US.

### Case 2: Louise

Louise is a 16-year-old female who was diagnosed with Autism Spectrum Disorder (ASD). She was determined to function at the cognitive level of a 13-year-old child. Louise had been raised in a small rural village in Italy by her biological parents up until the age of nine when her parents divorced.

When Louise was 11, her mother consented to a holiday in which Louise traveled to Canada with her father. While in Canada, Louise's father enrolled her in school, arranged psychological treatment for her ASD, as well as psychiatric treatment for comorbid conditions including obsessive-compulsive disorder (OCD).

Louise's mother attempted to have her returned to Italy over the course of 3 years. After multiple failed mediation attempts, she pursued an application under the Hague Convention. Over the course of 5 years that Louise remained in Canada, she had connected with various mental health workers and attended school with special modifications to her programming.

Louise was appointed legal counsel and a forensic psychiatric assessment was requested. Over the course of the assessment, Louise made various allegations regarding emotional abuse toward her by her mother. Louise stated that when she resided in Italy she would be belittled by her mother and was called various derogatory words that were translated to mean “retard” or “mongrel.”

The psychiatric opinion concluded that Louise may suffer potential harm should she be returned to Italy. The nature of her underlying OCD was explored; it was determined that she would have reduced access to child and adolescent psychiatric services and the disruption of her environment would likely exacerbate her symptoms. Furthermore, it was noted that Louise had been provided with significant educational modifications while residing in Canada, which may not have been available upon her return to a rural setting in Italy.

Louise was ordered to be returned to Italy given that she was determined to not meet criteria for grave significant harm should she be placed under the care of her mother.

## Discussion

These case examples elucidate various challenges which arise in the context of psycholegal assessments related to ICA and the Hague Convention. In both cases, the children were determined to not meet criteria set forth under Article 13 of the Convention and were ordered to be returned to their countries of origin. In reaching their decisions, judges referenced the existence of legislative and judicial instruments to ensure the safety and well-being of the children in their respective home countries.

**Figure 2:**
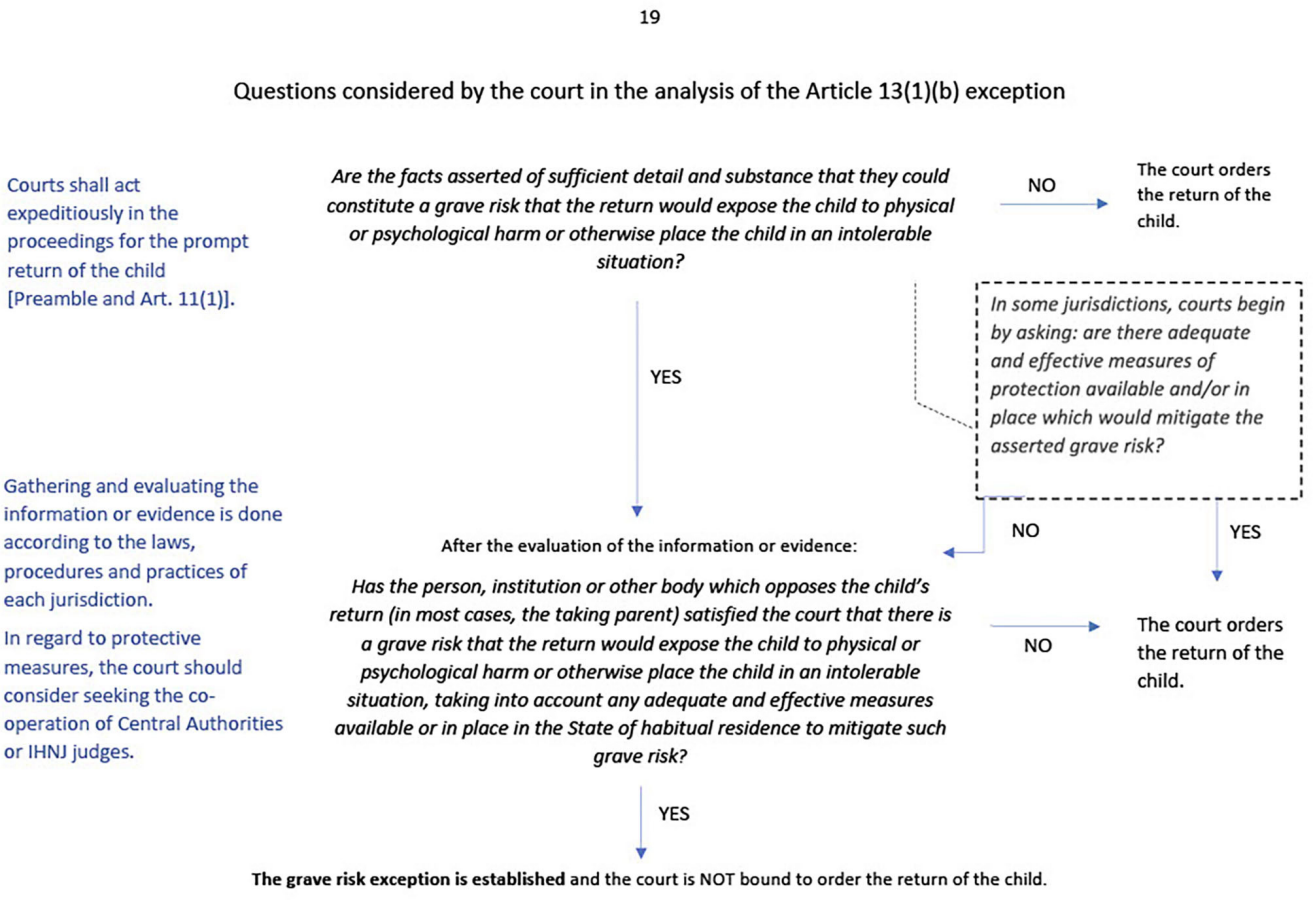
Decision making process related to grave risk exception. Retrieved from http://www.hcch.net on May 15, 2020.

When attempting to determine grave risk, the courts must consider several factors. These include whether a grave risk exists, the probability of that grave risk affecting the child, and whether adequate resources are in place to mitigate said risk. An example of a court's decision-making process relating to Article 13(1)(b) is summarized in Figure 2.

The manner in which certain criteria are met to order the return of a child is not clearly defined by the Hague Convention directly. Various cases explore how these criteria are met and can inform the manner in which psycholegal opinions are sought and provided. Three specific areas that have been explored are: (1) how the views of the child are weighted or considered; (2) the nature of risk that can befall the child; and (3) the definition of “habitual residence.”

### How the Views of the Child Are Weighed or Considered

While the Hague Convention does not explicitly provide an avenue by which a child may participate in the hearing, case law provides various examples of successful appeals regarding the Hague Convention when a child's rights were deemed not to have been upheld.

For instance, in the Ontario Court of Appeal case of AMRI v KER (10), it was determined that meaningful procedural protections were not offered or afforded to the child resulting in a successful appeal of a decision to return the child to their home country. This case identified various rights of the child in applications before the Hague Convention; specifically, these rights include receiving notice of application and adequate disclosure, having a reasonable opportunity to respond, having their views considered in accordance with age and maturity and a right to legal representation.

While capacity may be assessed and defined differently among jurisdictions, a general approach includes providing an explanation of the nature of the illness and proposed treatment, the risks and anticipated benefits of said treatment, the existence and potential benefits of alternative treatments which includes the option of no treatment (11). In the case of children before the Hague Convention, expert assessments can assist to assess capacity regarding court processes, degree of maturity, and implications concerning which parent a child chooses to live with.

### The Nature of Risk That Can Befall the Child

When discussing risk, the nature can be physical, psychological, or otherwise place the child in an intolerable situation. If the nature is physical, there are often tangible pieces of evidence including photographs, hospital reports, and radiographic images. These evidentiary examples are perhaps more challenging to refute due to the physical nature of the evidence. In contrast, psychological harm and the extent of its severity is open to greater degrees of interpretation. Cases have attempted to define what psychological harm is.

In the case of Thomson v Thomson, further clarity is provided as to the nature of risk that is to be considered in exercising a return of a child under the Convention (12). This case identified that “…the risk has to be more than an ordinary risk… that not only must the risk be a weighty one, but that it must be one of substantial, and not trivial, psychological harm. That… is the effect of the words ‘or otherwise place the child in an intolerable situation”' (pg. 597b).

### Definition of ‘Habitual Residence'

In the case of the Office for Children's Lawyer v Balev (13), the court attempted to delineate what is meant by “habitual residence.” To determine habitual residence, this case points to a hybrid approach, requiring a court to look at the child's links and circumstances related to country A, the circumstances of the move from country A to country B, and the child's links and circumstances in country B. Considerations include duration of stay, conditions, and the child's preference. If the child objects to the return, or if the child has reached an appropriate age and degree of maturity, then the views of the child can be taken into account and potentially determine the country of “habitual residence.”

Expert opinion may be sought in relation to whether a child's preferences regarding “habitual residence” are unduly influenced by external factors, such as pressure by one parent vs. another. Furthermore, the ability for the child to adapt to a new environment may also play a role in their perspective of what is their “habitual residence.”

### Expert Psycholegal Opinions

The extent to which professional opinions are relied upon by courts or other administrative bodies remains an area of considerable debate. As evidenced in case one above, an opinion regarding the outcome and nature of a forensic psychiatric opinion was challenged without direct evaluation of the child by a RSW.

Any diagnoses offered by way of psycholegal assessment should be completed by a qualified professional with sufficient experience and expertise. In Canada, there is case law on who is an expert (14). Additionally, any diagnostic entity proposed should, if possible, involve the direct assessment of the individual in question. Vulnerable children are reliant on the individuals and processes to ensure their safety and well-being, particularly in the context of judicial proceedings.

Further criteria for mental health evaluations in relation to these forms of proceedings must be developed to ensure standards of practice. Whether an opinion can be provided without direct assessment of a child is questionable and raises ethical concerns regarding the manner in which opinions may be offered in the context of ICA.

If expert testimony is requested, courts should identify the specific question(s) being asked, remind parties of the limited scope of the proceedings, and select an appropriate expert to ensure the question can be answered by a qualified professional. Furthermore, as per the Canadian Association of Psychiatry and the Law, every effort should be made to directly interview the individual for whom an assessment is provided (15). Finally, diagnostic formulations should be consistent with widely accepted mental health diagnoses.

### Parental Alienation Syndrome

The “diagnostic entity” of PAS is questionable (16) and is currently not included in the DSM-5. Others, however, argue that PAS can be included within the DSM-5 as a “Parent-Child Relational Problem” (17). The Supreme Court of Canada has accepted the so called Daubert criteria, which has been used in the United States, in Canada for the admissibility of controversial scientific concepts in an expert opinion (18). In certain proceedings there is a *voir dire* to determine the admissibility of certain types of expert evidence. Glancy and Regehr (19) analyze this issue in greater depth.

While it is important to recognize that alienating parents, usually those with custody of the child (20), can unduly influence their children, it is imperative that an objective opinion be provided based upon a fulsome evaluation of the child. Such an evaluation should determine whether the child is a “mature minor” and capable of appreciating the consequences of various decisions, and the manner in which they come to such decisions.

### Forensic Psychiatric Assessments

To assist in determining the effects of abduction on a child, a child's legal counsel may request a forensic psychiatric assessment. This assessment may provide opinions regarding the potential for harm to befall the child, further findings regarding maturity and capacity to engage in courtroom proceedings, psychiatric diagnoses and potential interventions, which may improve the child's mental health.

The determination of psychological and physical harm to the child should be based upon a meticulous review of the available information and direct assessment of the child and parents when possible.

Finally, there are ethical dilemmas regarding the determination that direct harm may befall a child should they be returned to another country and parent. While identifying specific harm and highlighting risk are important, a child or forensic psychiatrist may be faced with ethical questions regarding their duty to report based upon their home country of practice. This can involve significant complexities regarding reporting criteria to other agencies across international boundaries.

## Conclusion

The Hague Convention provides an important mechanism to return children to their home country. As made evident by both cases examples provided, there are multiple psycholegal issues which can arise in the context of applications before the Hague Convention. Forensic psychiatric assessments can provide an objective lens which incorporates a child's perspectives, psychiatric diagnoses and therapeutic recommendations. It is imperative that such evaluations involve a direct assessment of the child and are completed by competent mental health professionals.

## Data Availability Statement

The original contributions generated for this study are included in the article/supplementary material, further inquiries can be directed to the corresponding author/s.

## Ethics Statement

Written informed consent was obtained from the minor(s)' legal guardian/next of kin for the publication of any potentially identifiable images or data included in this article.

## Author Contributions

All authors listed have made a substantial, direct and intellectual contribution to the work, and approved it for publication.

## Conflict of Interest

The authors declare that the research was conducted in the absence of any commercial or financial relationships that could be construed as a potential conflict of interest.
